# Tracheal Tube Obstruction Due to Hemoptysis Associated With Pulmonary Infarction in a Patient With Severe COVID-19 Pneumonia

**DOI:** 10.7759/cureus.13599

**Published:** 2021-02-27

**Authors:** Takaaki Maruhashi, Tatsuhiko Wada, Tomonari Masuda, Kunihiro Yamaoka, Yasushi Asari

**Affiliations:** 1 Department of Emergency and Critical Care Medicine, Kitasato University School of Medicine, Sagamihara, JPN; 2 Department of Rheumatology and Infectious Diseases, Kitasato University School of Medicine, Sagamihara, JPN

**Keywords:** covid-19, pulmonary embolism (pe), case report

## Abstract

The incidence of thrombotic complications is extremely high among severe coronavirus disease 2019 (COVID-19) patients in the intensive care unit. Various factors such as a cytokine storm due to an excessive immune response to inflammation, hypoxemia, and disseminated intravascular coagulation are considered predisposing factors for thrombotic complications.

A 55-year-old Japanese man intubated eight days previously was referred to our hospital because of a severe COVID-19 pneumonia diagnosis after his pharyngeal swab tested positive for severe acute respiratory syndrome coronavirus 2 using reverse transcription-polymerase chain reaction. The patient continued to remain hypoxic (PaO_2_/FiO_2_ ratio <100 mmHg) at the referring hospital. On admission, we initiated veno-venous extracorporeal membrane oxygenation (VV-ECMO). Unfractionated heparin and nafamostat mesylate were used as anticoagulants during VV-ECMO. Despite adequate anticoagulant therapy, he developed pulmonary infarction due to pulmonary embolism followed by hemoptysis. On day 10 following admission, his oxygen saturation dropped from 95% to 88%, with a marked decrease in his ventilator tidal volume, accompanied by an inability to ventilate the patient. Thereafter, we increased the VV-ECMO flow and exchanged his endotracheal tube. The lumen of the removed tracheal tube was found to be occluded by a large-sized blood coagulum. There was no further episode of tube occlusion. The patient was discharged in a walkable state on day 39 following admission.

Endotracheal tube obstruction secondary to hemoptysis should be suggested in patients with COVID-19 requiring ventilator support, as they are unable to perform frequent endotracheal tube suctions owing to the risk of infection.

## Introduction

The coronavirus disease 2019 (COVID-19) pandemic is yet to show any sign of convergence. COVID-19 causes acute respiratory distress syndrome, and the virus may injure not only the lungs but also various other organs, such as the myocardium [1], kidney [2,3], and pancreas [4]. In addition, some recent reports have revealed the risk of embolism in severe COVID-19 cases, which is independent of the state of predisposing thromboembolic risk [5-8]. Herein, we report a rare complication of tracheal tube obstruction in a patient with severe COVID-19 pneumonia and pulmonary embolism. This article was previously posted to the Research Square preprint server on September 15, 2020.

## Case presentation

**Figure 1:**
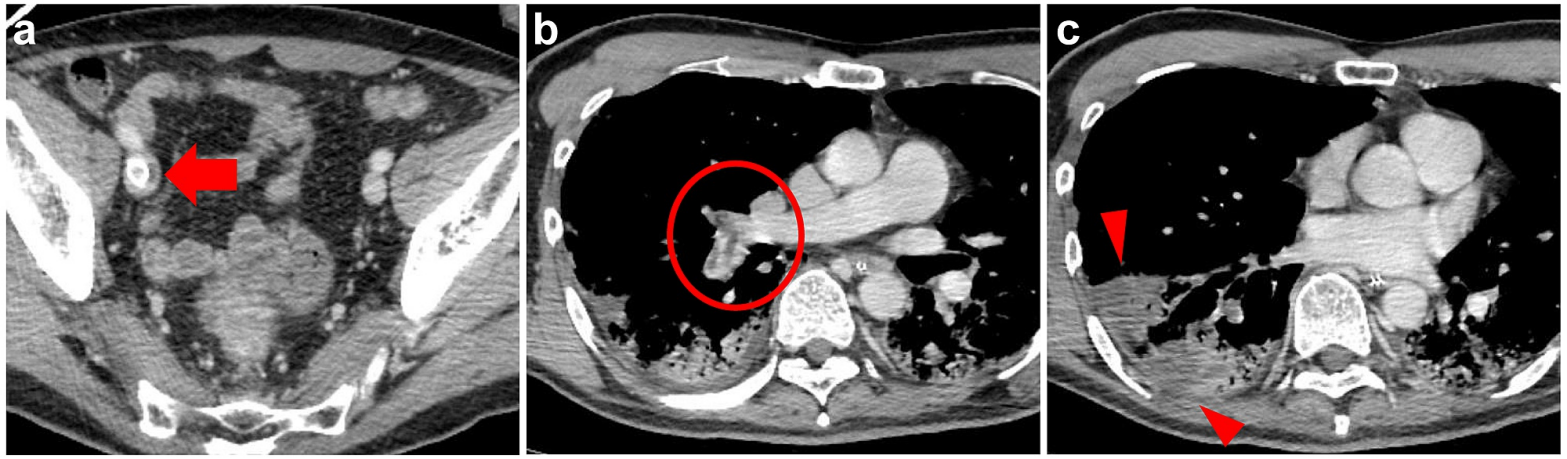
Chest-pelvis contrast-enhanced computed tomography images (a, b) Deep vein thrombosis of the right iliac vein around the drainage cannula for extracorporeal membrane oxygenation (arrow) and pulmonary embolism of the right main pulmonary artery (circle). (c) Pulmonary infarction, most likely secondary to a pulmonary embolism owing to the presence of poor contrast over a wedge along the embolized pulmonary artery region (arrowhead).

A 55-year-old Japanese man was referred to our hospital because of a severe COVID-19 pneumonia diagnosis after his pharyngeal swab tested positive for severe acute respiratory syndrome coronavirus 2 using reverse transcription-polymerase chain reaction, eight days following intubation for persistent hypoxia (PaO2/FiO2 ratio <100 mmHg). He had no history of smoking or any other underlying comorbidity, including any predisposition to thrombosis. The presence of thrombus was not confirmed at the time of admission to our hospital, even though antithrombotic therapy was not performed at the referral hospital. We initiated the patient on veno-venous extracorporeal membrane oxygenation (VV-ECMO) for providing respiratory support. During VV-ECMO, unfractionated heparin (UFH) 10000-15000 units/day and nafamostat mesylate at a fixed infusion rate of 30 mg/h were provided. Activated partial thromboplastin time (APTT) was maintained in the therapeutic range of 40-60 seconds. Despite adequate anticoagulant therapy, his D-dimer level was found to be elevated (87.2 µg/mL). Pulmonary embolism involving the right main pulmonary artery and right iliac vein thrombosis around the ECMO cannula insertion site in the right femoral vein was confirmed using contrast-enhanced computed tomography (CECT) (Figures 1a, 1b). CECT also identified a wedge-shaped infiltrate of poor contrast enhancement along the embolized pulmonary artery, consistent with pulmonary infarction (Figure 1c). The patient produced bloody sputum, most likely owing to pulmonary infarction.

**Figure 2:**
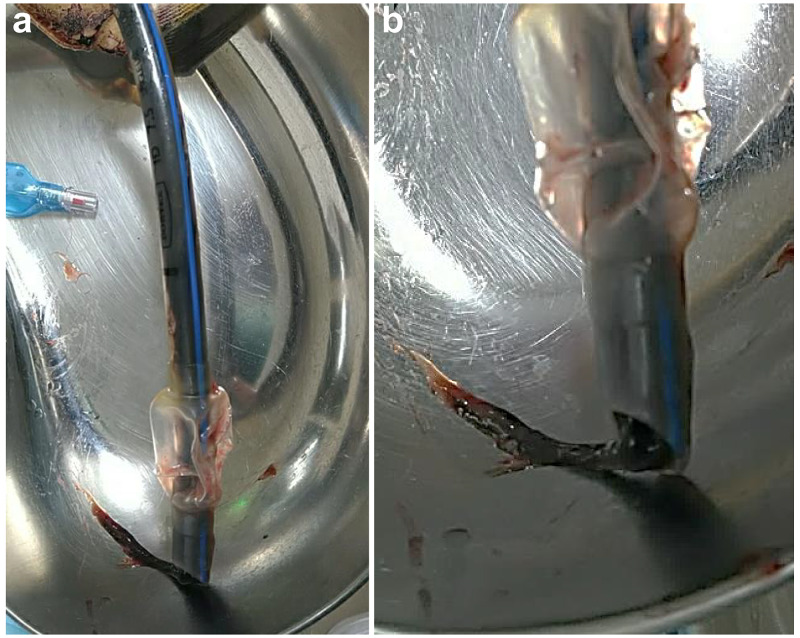
Removed tracheal tube (a) Total length of the tracheal tube indicates the size of the coagulum. (b) Enlarged image of the tracheal tube tip. On day 10, the patient’s tidal volume and saturation dropped to extremely low levels. A tracheal tube exchange was performed, and ventilation failure resolved. The tube lumen was mostly occluded by blood coagulum.

Nursing care, including endotracheal tube suctions during VV-ECMO, was performed at regular intervals. On day 10 following admission, the patient’s oxygen saturation dropped from 95% to 88%, with a marked reduction in his ventilator tidal volume, accompanied by an inability to ventilate the patient. On attempting endotracheal suctioning, the suction tube did not pass through the endotracheal tube. In response to this situation, we increased the VV-ECMO flow and performed an endotracheal tube exchange. We found that the lumen of the endotracheal tube was occluded by a large-sized blood coagulum (Figure 2).

Subsequently, nafamostat mesylate was discontinued, only UFH was continued, and a tracheotomy was performed. There was no further episode of tube occlusion. The patient was taken off VV-ECMO on day 10 and discharged in a walkable state on day 39 of admission.

## Discussion

The incidence of thrombotic complications among severe COVID-19 patients in intensive care units (ICU) is extremely high (31%) [6]. The predisposing factors for thrombotic complications in COVID-19 patients include various factors such as a cytokine storm due to an excessive immune response to inflammation, hypoxemia, and disseminated intravascular coagulation [9-12].

In our case, the patient was initiated on VV-ECMO and received UFH. The UFH dose was titrated carefully based on APTT monitoring. Our patient developed pulmonary embolism despite these measures. A pulmonary embolism leads to pulmonary infarction, as in this case, and among patients with pulmonary infarction, 13% develop hemoptysis [13]. The complication of endotracheal tube block by blood coagulum may be explained by the coexistence of hypercoagulability and bleeding tendency against the background of COVID-19 pneumonia. Therefore, severe COVID-19 patients may require careful monitoring of blood coagulation status and devising antithrombotic therapy.

We also considered other possible causes of endotracheal tube obstruction. The risk of health care workers (HCWs) acquiring an infection, as they are involved in the care of patients with COVID-19, is approximately 1.1%. This risk may increase during tracheal tube insertion and endotracheal suctioning, procedures known to generate aerosols [14]. Consequently, it is possible that frequent endotracheal tube suctioning, as is normally recommended in the ICU management of intubated patients with severe COVID-19 pneumonia, may not have been performed in this case. Measures to minimize the risk of an HCW acquiring an infection while performing endotracheal suctioning are warranted as well as increasing their confidence in adhering to the required protocols. This may reduce the incidence of endotracheal tube blockage among patients managed on ventilators. Fortunately, in our case, we could exchange the obstructed tracheal tube because of the respiratory assistance provided by VV-ECMO. However, in the absence of VV-ECMO, urgent measures are required for a rapid exchange of endotracheal tube.

## Conclusions

In patients with severe COVID-19 requiring ventilator support, a high suspicion of endotracheal tube obstruction secondary to various factors should be maintained to avoid adverse outcomes.
